# Association of triglyceride–glucose index and traditional risk factors with cardiovascular disease among non-diabetic population: a 10-year prospective cohort study

**DOI:** 10.1186/s12933-022-01694-3

**Published:** 2022-11-24

**Authors:** Li Liu, Zhenguo Wu, Yifan Zhuang, Yerui Zhang, Huiliang Cui, Fanghong Lu, Jie Peng, Jianmin Yang

**Affiliations:** 1 The Key Laboratory of Cardiovascular Remodeling and Function Research, Chinese Ministry of Education, Chinese National Health Commission and Chinese Academy of Medical Sciences, The State and Shandong Province Joint Key Laboratory of Translational Cardiovascular Medicine, Department of Cardiology, Qilu Hospital of Shandong University, No 107, Wenhuaxi Road, Jinan, Shandong China; 2 Cardio-Cerebrovascular Control and Research Center, Shandong Academy of Medical Sciences, Jinan, China; 3 Department of Geriatric Medicine, Key Laboratory of Cardiovascular Proteomics of Shandong Province, Qilu Hospital of Shandong University, No 107, Wenhuaxi Road, Jinan, China

**Keywords:** The TyG index, Cardiovascular disease, Coronary heart disease, Stroke, Eastern China

## Abstract

**Background:**

The triglyceride–glucose (TyG) index is known as a reliable alternative marker of insulin resistance (IR), which has been regarded as a predictor of cardiovascular disease (CVD). However, whether TyG index can predict the risk and occurrence of CVD in non-diabetic population remains uncertain. The aim of this study was to explore the association between the TyG index and cardiovascular risk factors and to clarify the prognostic value of the TyG index for CVD, coronary heart disease (CHD) and stroke in non-diabetic general population in Eastern China.

**Methods:**

A total of 6095 cases without diagnosed diabetes and CVD were included. The TyG index was calculated as ln (fasting triglyceride [mg/dL] × fasting glucose [mg/dL]/2) and the participants were divided into 4 groups according to the TyG index quartiles (Q1, Q2, Q3, Q4). The primary outcome was CVD, including CHD and stroke. Cox proportional hazards regression analysis was used to investigate the association between the TyG index and the risk of CVD.

**Results:**

During the 10-year follow-up, 357 (5.9%) participants of CVD, 224 (3.7%) participants of CHD and 151 (2.5%) participants of stroke were observed. The incidence of CVD increased with the TyG index quartiles. Multivariate Cox regression analysis showed that the hazard ratios [95% confidence interval (CI)] in Q4 group were respectively 1.484 (1.074–2.051) for CVD, 1.687 (1.105–2.575) for CHD and 1.402 (0.853–2.305) for stroke compared to Q1 group. Moreover, adding the TyG index to models with traditional risk factors yielded a significant improvement in discrimination and reclassification of incident CVD and CHD.

**Conclusions:**

The TyG index is associated with cardiovascular risk factors and can be used as a useful, low-cost predictive marker for CVD and CHD risk in non-diabetic population.

**Supplementary Information:**

The online version contains supplementary material available at 10.1186/s12933-022-01694-3.

## Background

Cardiovascular disease (CVD) accounts for the largest proportion of death causes among all global burden of diseases [1]. It is necessary to make better CVD risk prediction because the traditional model classification of cardiovascular risk factors is not enough for more and more CVD patients now in clinical practice [2]. A growing body of evidences showed that insulin resistance (IR) played a critical role in the pathogenesis of diabetes as well as CVD. Although the hyperinsulinaemic–euglycaemic clamp is considered as the gold standard diagnostic method for IR, this technique is not commonly used in large epidemiological investigations due to the complex testing process and expensive cost. Recently, the triglyceride–glucose (TyG) index has been considered as a time-saving, low-cost and relatively simple marker for IR [3].There were some researches investigating the positive relationship of the TyG index with CVD. Studies in Korea demonstrated the TyG index could be a useful marker to predict CVD in participants aged > 40 years [2] and in young adults aged 20–39 years [4]. Guo W et al. reported that the TyG index was independently associated with arterial stiffness and 10-year CVD risk evaluated using Framingham risk score among a sample of apparently healthy Chinese population [5]. The Kailuan study in Northern China also evidenced the positive association between the TyG index and cardiovascular diseases [6].

China is one of the two countries with the highest burdens of cardiovascular diseases among the world and CVD has been the most important cause of death in China, accounting for more than 40% of deaths in Chinese population [7]. However, few studies on CVD risk prediction by the TyG index were developed in Eastern Chinese population. What is more, the study by Alizargar J et al. showed that the use of the TyG index for diagnosing and predicting cardiovascular diseases in patients with CVD and diabetes might be biased and had lower value than expected [8]. However, the relation between the TyG index and CVD in general non-diabetic population is uncertain. Considering the different effects of epidemiological and metabolic risk factors in different population and the different lifestyles in different regions [9], we aimed to investigate the association of the TyG index with cardiovascular risk factors and the risk of CVD, CHD and stroke in a community-based prospective non-diabetic cohort in Eastern China.

## Methods

### Study population

**Fig. 1:**
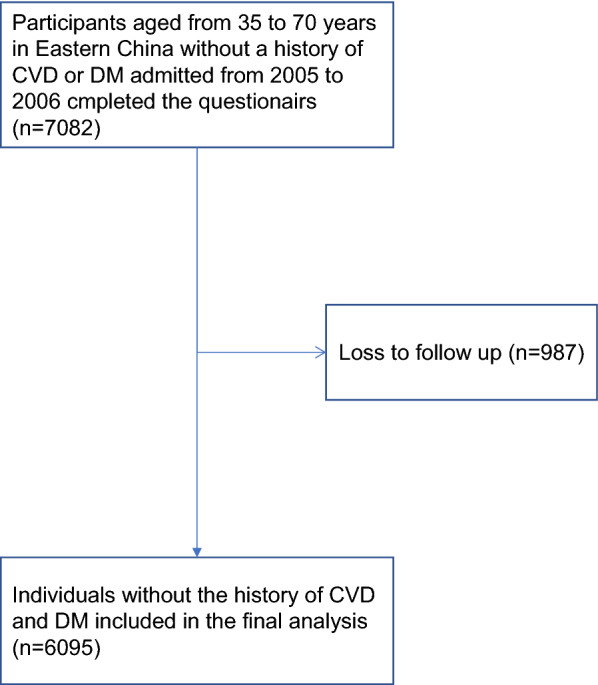
Flow diagram of patient selection. CVD, Cardiovascular disease; CHD, Coronary heart disease

From 2005 to 2006, we collected data of 12 communities aged 35 to 70 years who had lived in Eastern China for at least five years through a random, multi-stage and cluster sampling scheme. A total of 7082 participants without a history of CVD or diabetes mellitus (DM) completed the questionnaires. 987 individuals were lost to follow-up, and a total of 6095 individuals involving 2990 men (49.1%) and 3105 women (50.9%) were included in this study (Fig. 1).

This study was approved by the Ethics Review Committee of the Shandong Academy of Medical Sciences (Approval No. 202111120194) and complied with the Declaration of Helsinki. Written informed consent were provided from all participants.

### Data collection and definitions

Standardized questionnaire by trained staffs were used to collect information of participants about clinical and demographic characteristics including age, sex, medication, lifestyle and education. Education level was classified as primary or less, secondary, and trade, college or university. Tobacco and alcohol use were classified as never, former and current. Energy intake from fat and carbohydrate were split into tertiles (Energy intake from fat: tertile 1, ≤ 13.41%; tertile 2, 13.41% to 17.74%; tertile 3, > 17.74%. Energy intake from carbohydrate: tertile 1, ≤ 59.72%; tertile 2, 59.72% to 68.43%; tertile 3, > 68.43%). Physical activity was assessed by the International Physical Activity Questionnaire (IPAQ)[10] and low physical activity was defined as < 600 metabolic equivalent task (MET)$\times$ minutes per week or < 150 min per week of moderate intensity physical activity. Body mass index (BMI) and waist hip ratio (WHR) were calculated respectively by the 2 formulas: weight(kg)/height(m)^2^ and waist circumference(cm)/hip circumference(cm). Hypertension was defined as SBP ≥ 140 mmHg or DBP ≥ 90 mmHg. Obesity was defined as BMI ≥ 30 kg/m^2^ as previous described [11]. Before blood collection, participants were forbidden to drink and exercise violently within 24 h and were forbidden to change their eating habits. EDTA anticoagulation tubes were used to collect blood sample from venous blood of the anterior elbow after at least eight-hour fasting in the morning at 7 to 9 o’clock. Then these blood samples were sent to the laboratory for testing including fasting plasma glucose (FPG), total cholesterol (TC), triglyceride (TG) and high-density lipoprotein cholesterol (HDL-C) using an autoanalyzer. Low-density lipoprotein cholesterol (LDL-C) was estimated using Friedewald’s formula [12]. Blood pressure (BP) was measured by the sphygmomanometer according to standard steps with no smoking and no coffee for half an hour and resting in a quiet environment for at least 5 min.

The TyG index was calculated by the formula [ln (fasting triglyceride [mg/dL] × fasting glucose [mg/dL]/2)] [13]. The primary endpoint was CVD and the secondary endpoints were coronary heart disease (CHD) and stroke. CHD was defined as myocardial infarction, angina pectoris and angiography-proven coronary heart disease, and stroke included ischemic, hemorrhagic, or unspecified stroke. CVD was defined as the composite of CHD and stroke.

### Statistical analysis

SPSS version 25.0 (SPSS, Chicago, IL) and R software version 4.2.0 (R Foundation for Statistical Computing) were used for statistical analysis. Continuous variables were described by mean and standard deviation (SD) and categorical variables were described by frequency and percentage. One-way ANOVA for continuous variables and the Pearson chi-square test for categorical variables were used for the comparison of baseline data of different TyG quartile groups. Pearson or Spearman correlation analysis was used to evaluate the association between the TyG index and cardiovascular risk factors. During the total follow-up period, the incident rates of the three outcomes (CVD, CHD, stroke) for each TyG quartile group were calculated. Kaplan–Meier analysis was used to draw the incidence curves by R language and the Log-rank test was used for the *p* value. In order to clarify the independent predictive value of the TyG index, we developed multivariate Cox proportional hazards regression and controlled the confounding factors by building 6 regression models. Model 1 was unadjusted; Model 2 was adjusted for age and gender; Model 4 was fully adjusted for age, gender, WHR, tobacco use, alcohol use, education, physical activity, hypertension, BMI, LDL-C, intake of fat and carbohydrates, use of antihypertensive drugs and antilipemic drugs. Moreover, we used these fully adjusted variables to develop multivariate Cox regression analyses for the incidence of CVD, CHD and stroke. And Model 3, 5, 6 were respectively partially adjusted for the variables statistically associated with the incidence of CVD, CHD and stroke. *P* value less than 0.05 was considered to be statistically significant.

## Results

### Baseline characteristics of the study participants according to the TyG quartiles

Table 1 showed the baseline characteristics of the 6095 participants in the cohort study according to the TyG quartiles. Among them, the average age of the participants was 48.69 ± 9.95 years, and 49.1% were men. Individuals in the higher TyG index quartile were more likely to be with current alcohol use, obesity, hypertension, use of antihypertensive drugs and to have a higher age, BMI, WHR, SBP, DBP, FPG, TC and TG (all *p* ≤ 0.001). And the differences of LDL, HDL, sex, education levels, the intake of fat and carbohydrates in the four groups were significant (all *p* < 0.01). In terms of endpoints, significantly increased prevalence of CVD and CHD was found in the Q4 group than in the other groups (all *p* < 0.001) (Table 1).

**Table 1:** Baseline characteristics of the study population according to the TyG quartiles

Variables	TyG index				*p*-value	Total (n = 6095)
	Quartile 1	Quartile 2	Quartile 3	Quartile 4		
	(n = 1514)	(n = 1514)	(n = 1543)	(n = 1524)		
TyG index	≤ 7.92	> 7.92 to ≤ 8.32	> 8.32 to ≤ 8.76	> 8.76		
Age(years)	45.87 ± 9.79	48.18 ± 9.97	49.98 ± 9.77	50.70 ± 9.59	** < 0.001**	48.69 ± 9.95
Gender, n (%)						
Male	707 (46.7)	732 (48.3)	737 (47.8)	814 (53.4)	**0.001**	2990 (49.1)
Female	807 (53.3)	782 (51.7)	806 (52.2)	710 (46.6)		3105 (50.9)
BMI (kg/m^2^)	22.87 ± 3.22	23.93 ± 3.37	25.11 ± 3.21	26.13 ± 3.41	** < 0.001**	24.51 ± 3.52
WHR	0.86 ± 0.05	0.87 ± 0.05	0.87 ± 0.04	0.88 ± 0.05	** < 0.001**	0.87 ± 0.05
SBP (mm Hg)	132.71 ± 20.80	135.96 ± 20.61	140.13 ± 22.67	142.64 ± 21.68	** < 0.001**	137.88 ± 21.79
DBP (mm Hg)	80.40 ± 12.43	82.93 ± 12.42	85.43 ± 12.97	87.68 ± 12.41	** < 0.001**	84.12 ± 12.85
Tobacco use, n (%)						
Never	1174 (77.5)	1158 (76.5)	1188 (77.0)	1159 (76.0)	0.537	4679 (76.8)
Former	38 (2.5)	33 (2.2)	49 (3.2)	38 (2.5)		158 (2.6)
Current	302 (19.9)	323 (21.3)	306 (19.8)	327 (21.5)		1258 (20.6)
Alcohol use, n (%)						
Never	1218 (80.4)	1199 (79.2)	1215 (78.7)	1130 (74.1)	**0.001**	4762 (78.1)
Former	17 (1.1)	22 (1.5)	29 (1.9)	29 (1.9)		97 (1.6)
Current	279 (18.4)	293 (19.4)	299 (19.4)	365 (24.0)		1236 (20.3)
Low physical activity	245 (16.2)	234 (15.5)	239 (15.5)	258 (16.9)	0.649	976 (16.0)
Education, n (%)						
Primary or less	541 (35.7)	558 (36.9)	581 (37.7)	561 (36.8)	**0.001**	2241 (36.8)
Secondary	899 (59.4)	868 (57.3)	834 (54.1)	842 (55.2)		3443 (56.5)
Trade, college or university	74 (4.9)	88 (5.8)	128 (8.3)	121 (7.9)		411 (6.7)
Hypertension, n (%)	511 (33.8)	658 (43.5)	793 (51.4)	870 (57.1)	** < 0.001**	2832 (46.5)
Obesity, n (%)	32 (2.1)	58 (3.8)	102 (6.6)	180 (11.8)	** < 0.001**	372 (6.1)
FPG (mmol/L)	4.05 ± 0.67	4.37 ± 0.68	4.62 ± 0.72	4.89 ± 0.76	** < 0.001**	4.48 ± 0.77
TC (mmol/L)	4.09 ± 0.77	4.51 ± 0.80	4.78 ± 0.84	5.09 ± 1.01	** < 0.001**	4.62 ± 0.94
TG (mmol/L)	0.65 ± 0.15	1.00 ± 0.19	1.41 ± 0.27	2.91 ± 1.76	** < 0.001**	1.49 ± 1.24
LDL-C (mmol/L)	2.52 ± 0.64	2.76 ± 0.66	2.85 ± 0.68	2.61 ± 0.92	** < 0.001**	2.69 ± 0.74
HDL-C (mmol/L)	1.26 ± 0.36	1.28 ± 0.30	1.29 ± 0.28	1.25 ± 0.30	** < 0.001**	1.27 ± 0.31
Antihypertensive drugs,n (%)	88 (5.8)	121 (8.0)	177 (11.5)	223 (14.6)	** < 0.001**	609 (10.0)
Antilipemic drugs, n (%)	26 (1.7)	28 (1.9)	39 (2.5)	39 (2.6)	0.239	132 (2.2)
FAT, n (%)						
Tertile 1	533 (35.2)	516 (34.1)	484 (31.4)	501 (32.9)	**0.003**	2034 (33.4)
Tertile 2	521 (34.4)	500 (33.0)	545 (35.3)	462 (30.3)		2028 (33.3)
Tertile 3	460 (30.4)	498 (32.9)	514 (33.3)	561 (36.8)		2033 (33.4)
Carbohydrates, n (%)						
Tertile 1	434 (28.7)	490 (32.4)	514 (33.3)	594 (39.0)	** < 0.001**	2032 (33.3)
Tertile 2	539 (35.6)	521 (34.4)	518 (33.6)	454 (29.8)		2032 (33.3)
Tertile 3	541 (35.7)	503 (33.2)	511 (33.1)	476 (31.2)		2031 (33.3)
CVD	62 (4.1)	83 (5.5)	81 (5.2)	131 (8.6)	** < 0.001**	357 (5.9)
CHD	33 (2.2)	51 (3.4)	51 (3.3)	89 (5.8)	** < 0.001**	224 (3.7)
Stroke	29 (1.9)	38 (2.5)	36 (2.3)	48 (3.1)	0.174	151 (2.5)

Continuous variables were given as mean ± SD and categorical variables were given by frequency and percentage as n (%)

*TyG index* Triglyceride–glucose index; *BMI* body mass index; *WHR* Waist hip ratio; *SBP* Systolic blood pressure; *DBP* Diastolic blood pressure; *FPG* fasting plasma glucose; *TC* total cholesterol; *TG* triglyceride; *LDL-C* low-density lipoprotein-cholesterol; *HDL-C* high-density lipoprotein-cholesterol; *CVD* Cardiovascular disease; *CHD* Coronary heart disease

*p* values in bold are < 0.05

### Correlations between the TyG index and cardiovascular risk factors

The correlation between the TyG index and cardiovascular risk factors examined by Spearman or Pearson correlation analysis was displayed in Table 2. Among all participants, the TyG index was positively correlated to age (r = 0.198, *p* < 0.001), BMI (r = 0.342, *p* < 0.001), WHR (r = 0.178, *p* < 0.001), SBP (r = 0.176, *p* < 0.001), DBP (r = 0.211, *p* < 0.001) and TC (r = 0.420, *p* < 0.001). No significant correlation between the TyG index and LDL-C or HDL-C was observed (Table 2).

**Table 2:** Correlations between the TyG index and cardiovascular risk factors

Variables	Correlation coefficient	*p*-value
Age (years)	0.198^§^	** < 0.001**
BMI (kg/m^2^)	0.342^┼^	** < 0.001**
WHR	0.178^┼^	** < 0.001**
SBP (mmHg)	0.176^┼^	** < 0.001**
DBP (mmHg)	0.211^┼^	** < 0.001**
TC (mmol/L)	0.420^┼^	** < 0.001**
LDL-C (mmol/L)	-0.011^┼^	0.398
HDL-C (mmol/L)	-0.014^┼^	0.279

*TyG* *index* triglyceride–glucose index; *BMI* body mass index; *WHR* Waist hip ratio; *SBP* Systolic blood pressure; *DBP* Diastolic blood pressure; *TC* total cholesterol; *LDL-C* low-density lipoprotein-cholesterol; *HDL-C* high-density lipoprotein-cholesterol

*p* values in bold are < 0.05

^┼^Pearson correlation analysis

^§^Spearman correlation analysis

### Univariate Cox regression analyses for the incidence of CVD, CHD and stroke

The association of incidence CVD, CHD and stroke with covariates was shown in Additional file 1: Table S1. The covariates including age, tobacco use, alcohol use, education, physical activity, hypertension, BMI, intake of carbohydrates, use of antihypertensive drugs, and use of antilipemic drugs showed statistically significant association with the incidence CVD(*p* < 0.05). Likewise, age, tobacco use, physical activity, hypertension, BMI, LDL-C, intake of carbohydrates, use of antihypertensive drugs, and use of antilipemic drugs increased the risk of CHD incidence. Moreover, age, gender, tobacco use, alcohol use, education, hypertension, use of antihypertensive drugs, and use of antilipemic drugs were statistically significant related to the incidence stroke(*p* < 0.05). (Additional file 1: Table S1).

### Risk of CVD, CHD and stroke by the TyG quartiles

**Fig. 2:**
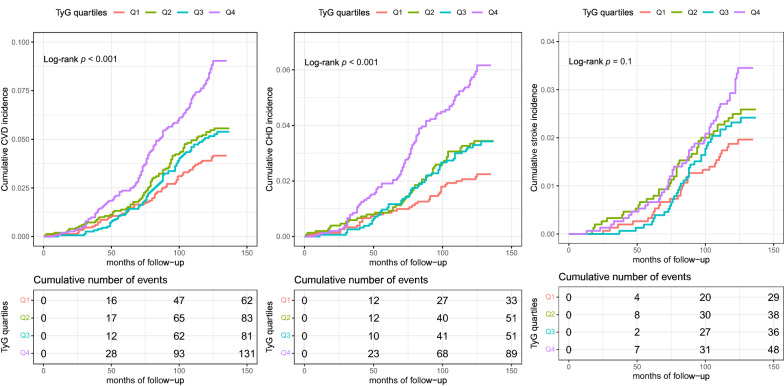
Kaplan–Meier curves of CVD (**A**), CHD (**B**), and stroke (**C**) by TyG index quartile. The cumulative incidence of CVD (**A**), CHD (**B**), and stroke (**C**) during follow-up grouped according to the TyG index quartile was analyzed by Kaplan–Meier curves. The *p* value was calculated with the log-rank test

Over a median follow-up of 10.58 years (interquartile range: 9.92 to 10.75), 357 (5.9%) CVD, 224 (3.7%) CHD and 151 (2.5%) stroke were identified. The Kaplan–Meier curves for the cumulative incidences of CVD, CHD and stroke grouped by the TyG index quartiles were shown in Fig. 2A–C. The probability of cumulative incidences of CVD and CHD was significantly higher in patients with a higher TyG index than in those with a lower TyG index (*p* < 0.001). Although the incidence of stroke was likely to increase with increasing quintiles of the TyG index, no statistical significance between the TyG index and incident stroke was found (Fig. 2).

As shown in Table 3, the hazard ratios (HR) (95% CI) calculated respectively in Model 1, 2, 3 and 4 for the incidence of CVD with per SD increase in the TyG index was 1.276 (1.159–1.405), 1.204 (1.087–1.334), 1.137 (1.019–1.269) and 1.145 (1.025–1.279) while with per unit increase in the TyG index was respectively 1.463 (1.259–1.702), 1.337 (1.140–1.568), 1.222 (1.030–1.450) and 1.236 (1.040–1.468). Compared with Group Q1, population in Group Q4 had a twofold higher risk of CVD in Model 1 [2.174 (1.607–2.941)]. The age- and sex-adjusted HR for the incidence of CVD increased by 70.7% (95%CI 1.260–2.311) in Group Q4. In partially adjusted Model 3 and fully adjusted Model 4, with the highest TyG index in Group Q4, the possibility of the incidence of CVD risk respectively increased by 43.5%[1.435 (1.040–1.981)] and 48.4%[1.484 (1.074–2.051)]. The increased risk of CVD from Group Q1 to Group Q4 in Model 1, 2, 3 and 4 was statistically significant (*p* for trend < 0.05).

**Table 3:** Univariate and Multivariate Cox regression analyses for incident CVD

TyG index	Incident CVD, n (%)	HR (95%CI)			
		Model 1	Model2	Model 3	Model 4
Per Unit increase	357 (5.9)	1.463 (1.259–1.702)^***^	1.337 (1.140–1.568)^***^	1.222 (1.030–1.450) ^*^	1.236 (1.040–1.468) ^*^
Per SD increase		1.276 (1.159–1.405)^***^	1.204 (1.087–1.334)^***^	1.137 (1.019–1.269)^*^	1.145 (1.025–1.279)^*^
Quartile 1	62 (4.1)	1 (Reference)	1 (Reference)	1 (Reference)	1 (Reference)
Quartile 2	83 (5.5)	1.352 (0.973–1.878)	1.185 (0.853–1.648)	1.106 (0.793–1.542)	1.125 (0.805–1.571)
Quartile 3	81 (5.2)	1.295 (0.930–1.802)	1.037 (0.744–1.445)	0.918 (0.653–1.290)	0.939 (0.666–1.324)
Quartile 4	131 (8.6)	2.174 (1.607–2.941)^***^	1.707 (1.260–2.311)^**^	1.435 (1.040–1.981)^*^	1.484 (1.074–2.051)^*^
*p* for trend		** < 0.001**	** < 0.001**	**0.011**	**0.008**

Model 1: unadjusted

Model 2: adjusted for age and gender

Model 3: adjusted for age, tobacco use, alcohol use, education, physical activity, hypertension, BMI, intake of carbohydrates, use of antihypertensive drugs, and use of antilipemic drugs

Model 4: adjusted for age, gender, WHR, tobacco use, alcohol use, education, physical activity, hypertension, BMI, LDL-C, intake of fat and carbohydrates, use of antihypertensive drugs, and use of antilipemic drugs

*CVD* cardiovascular disease; *TyG index* triglyceride–glucose index; *HR* hazard ratio; *CI* confidence interval; *SD* standard deviation

^**^p < 0.01

^***^p < 0.001

*p* values in bold are < 0.05

In Tables 4 and 5, with per SD increase in the TyG index, the fully adjusted HR (95%CI) for risk of CHD and stroke was respectively 1.188 (1.033–1.366) and 1.122 (0.946–1.330). Population in Group Q4 had an increased risk of the incidence for CHD [unadjusted HR 2.768 (1.856 to 4.127)] and stroke [unadjusted HR 1.706 (1.076 to 2.705)] compared to Group Q1. After adjustment for age and sex, population in Group Q4 had 2.2 times the risk of incidence of CHD compared to the population in Group Q1 (95%CI 1.453–3.243). After adjustment for partial covariates in Model 5 and 6, the risk in Group Q4 for the incidence of CHD and stroke was respectively 1.663 (1.091–2.534) and 1.298 (0.811–2.078). After adjustment for all covariates, population in Group Q4 had 1.687 times the risk of CHD incidence compared to the population in Q1 (95% CI 1.105–2.575). However, the increased risk of stroke from Group Q1 to Group Q4 in Model 1,2,3 and 4 showed no statistical significance (all *p* for trend > 0.05) (Tables 4, 5).

**Table 4:** Univariate and Multivariate Cox regression analyses for incident CHD

TyG index	Incident CHD, n (%)	HR (95%CI)			
		Model 1	Model 2	Model 5	Model 4
Per Unit increase	224 (3.7)	1.599 (1.326–1.928) ^***^	1.474 (1.209–1.798) ^***^	1.298 (1.045–1.612) ^*^	1.308 (1.052–1.627) ^*^
Per SD increase		1.350 (1.198–1.522) ^***^	1.282 (1.129–1.456) ^***^	1.182 (1.029–1.357) ^*^	1.188 (1.033–1.366) ^*^
Quartile 1	33 (2.2)	1 (Reference)	1 (Reference)	1 (Reference)	1 (Reference)
Quartile 2	51 (3.4)	1.559 (1.006–2.416) ^*^	1.372 (0.885–2.127)	1.222 (0.784–1.904)	1.225 (0.785–1.910)
Quartile 3	51 (3.3)	1.530 (0.988–2.371)	1.224 (0.788–1.900)	0.998 (0.635–1.567)	0.999 (0.635–1.570)
Quartile 4	89 (5.8)	2.768 (1.856–4.127) ^***^	2.171 (1.453–3.243) ^***^	1.663 (1.091–2.534) ^*^	1.687 (1.105–2.575) ^*^
*p* for trend		** < 0.001**	** < 0.001**	**0.014**	**0.011**

Model 1: unadjusted

Model 2: adjusted for age and gender

Model 5: adjusted for age, tobacco use, physical activity, hypertension, BMI, LDL-C, intake of carbohydrates, use of antihypertensive drugs, and use of antilipemic drugs

Model 4: adjusted for age, gender, WHR, tobacco use, alcohol use, education, physical activity, hypertension, BMI, LDL-C, intake of fat and carbohydrates, use of antihypertensive drugs, and use of antilipemic drugs

*CHD* coronary heart disease; *TyG index* triglyceride–glucose index; *HR* hazard ratio; *CI* confidence interval; *SD* standard deviation

^*^p < 0.05

^**^p < 0.01

^***^p < 0.001

*p* values in bold are < 0.05

**Table 5:** Univariate and Multivariate Cox regression analyses for incident stroke

TyG index	Incident stroke, n (%)	HR (95%CI)			
		Model 1	Model2	Model 6	Model 4
Per Unit increase	151 (2.5)	1.306 (1.030–1.656) ^*^	1.186 (0.924–1.523)	1.158 (0.900–1.490)	1.196 (0.917–1.561)
Per SD increase		1.186 (1.019–1.381) ^*^	1.116 (0.951–1.309)	1.098 (0.935–1.291)	1.122 (0.946–1.330)
Quartile 1	29 (1.9)	1 (Reference)	1 (Reference)	1 (Reference)	1 (Reference)
Quartile 2	38 (2.5)	1.324 (0.816–2.146)	1.160 (0.715–1.882)	1.153 (0.710–1.873)	1.207 (0.738–1.974)
Quartile 3	36 (2.3)	1.231 (0.755–2.008)	0.997 (0.611–1.628)	0.984 (0.600–1.615)	1.028 (0.617–1.710)
Quartile 4	48 (3.1)	1.706 (1.076–2.705) ^*^	1.356 (0.854–2.152)	1.298 (0.811–2.078)	1.402 (0.853–2.305)
*p* for trend		0.136	0.458	0.570	0.447

Model 1: unadjusted

Model 2: adjusted for age and gender

Model 6: adjusted for age, gender, tobacco use, alcohol use, education, hypertension, use of antihypertensive drugs, and use of antilipemic drugs

Model 4: adjusted for age, gender, WHR, tobacco use, alcohol use, education, physical activity, hypertension, BMI, LDL-C, intake of fat and carbohydrates, use of antihypertensive drugs, and use of antilipemic drugs

*TyG index* triglyceride–glucose index; *HR* hazard ratio; *CI* confidence interval; *SD* standard deviation

^*^p < 0.05

### Subgroup analysis

To explore the association between the TyG index and CVD, CHD and stroke incidence in detail, subgroup analyses stratified by sex, age, hypertension and BMI were performed. The association of the TyG index with the risk of CVD, CHD and stroke showed consistence and stability across subgroups.

In the light of baseline characteristics and cardiovascular risk factors, we classified the participants and calculated the HRs (95%CI) and *p* values for interaction of each group for CVD, CHD and stroke with fully adjusted in Model 4 in Table 6. We found that when cardiovascular risk factors not presented, the relationship between the TyG index and CVD, CHD, stroke was generally consistent. Subgroup analysis demonstrated higher predictive values of the TyG index for CVD, CHD and stroke in participants younger than 60 years, female, without hypertension or obesity. There was no significant interaction between the TyG index and sex, age, hypertension or BMI (all *p* > 0.05) (Table 6).

**Table 6:** Risk of CVD, CHD, and stroke according to a prespecified subgroup comparing the highest TyG index quartile with all others

Subgroup	CVD		CHD		Stroke	
	HR (95% CI)	*p* for interaction	HR (95% CI)	*p* for interaction	HR (95% CI)	*p* for interaction
Sex		0.232		0.313		0.316
Female	1.390 (1.072–1.802)^*^		1.480 (1.079–2.031)^*^		1.393 (0.915–2.121)	
Male	1.239 (0.972–1.580)		1.276 (0.930–1.751)		1.152 (0.803–1.654)	
Age		0.955		0.950		0.918
≤ 60	1.244 (1.016–1.522)^*^		1.307 (1.013–1.687)^*^		1.198 (0.878–1.634)	
> 60	1.150 (0.789–1.677)		1.308 (0.816–2.096)		1.073 (0.576–1.998)	
Hypertension		0.228		0.401		0.183
No	1.350 (1.007–1.810)^*^		1.470 (1.024–2.112)^*^		1.518 (0.951–2.424)	
Yes	1.190 (0.959–1.475)		1.228 (0.929–1.623)		1.098 (0.793–1.520)	
BMI		0.293		0.590		0.476
< 30	1.210 (1.007–1.455)^*^		1.268 (1.005–1.600)*		1.176 (0.884–1.563)	
≥ 30	1.499 (0.753–2.984)		1.197 (0.478–3.002)		1.449 (0.534–3.930)	

HR are adjusted for age, gender, WHR, tobacco use, alcohol use, education, physical activity, hypertension, BMI, LDL-C, intake of fat and carbohydrates, use of antihypertensive drugs, and use of antilipemic drugs

*CVD* Cardiovascular disease; *CHD*, Coronary heart disease; *TyG index*, triglyceride–glucose index; *HR*, hazard ratio; *CI* confidence interval

^*^p < 0.05

^**^p < 0.01

^***^p < 0.001

### Evaluation of the prognostic performance of the TyG index for CVD, CHD and stroke

As shown in Table 7, C-statistic, NRI and IDI were used to calculate the incremental predictive value of the TyG index for CVD, CHD and stroke and so as to evaluate the influence of the TyG index. We concluded that the addition of the TyG index into the baseline risk model, which contained age, gender, WHR, tobacco use, alcohol use, education, physical activity, hypertension, BMI, LDL-C, intake of fat and carbohydrates, use of antihypertensive drugs and antilipemic drugs, improved significantly the prediction probability for CVD, CHD and stroke. In light of C-statistic, after adding the TyG index, the prediction probability of the the baseline risk mode was improved significantly with C-statistic increased from 0.730 to 0.731 (*p* = 0.016) for CVD and C-statistic increased from 0.731 to 0.733 (*p* = 0.017) for CHD. Likewise, according to continuous NRI, the TyG index significantly improved the prediction for CVD [continuous NRI (95% CI) 0.1587(0.0518–0.2655), *p* = 0.004], CHD [continuous NRI (95%CI) 0.1637(0.0304–0.2971), *p* = 0.016] and stroke [continuous NRI (95%CI) 0.1818(0.0203–0.3432), *p* = 0.027]. However, IDI did not show statistically significance in improving the prediction of CVD, CHD and stroke. (Table 7).

**Table 7:** The incremental predictive value of the TyG index for CVD, CHD and stroke

CVD	C-statistic (95% CI)	*p*-value	Continuous NRI (95% CI)	*p*-value	IDI (95% CI)	*p*-value
Model 3 without TyG index	0.730 (0.706–0.754)	Ref		Ref		Ref
Model 3 with TyG index	0.731 (0.708–0.755)	**0.016**	0.1587 (0.0518—0.2655)	**0.004**	9e-04 (–3e–04–0.0021)	0.133
CHD						
Model 3 without TyG index	0.731 (0.700–0.762)	Ref		Ref		Ref
Model 3 with TyG index	0.733 (0.703–0.764)	**0.017**	0.1637 (0.0304—0.2971)	**0.016**	0.0012 (–1e–04—0.0025)	0.073
Stroke						
Model 3 without TyG index	0.758 (0.721–0.794)	Ref		Ref		Ref
Model 3 with TyG index	0.759 (0.723–0.795)	0.180	0.1818 (0.0203—0.3432)	**0.027**	2e-04 (–6e–04—0.001)	0.604

*CVD* Cardiovascular disease; *CHD* Coronary heart disease; *TyG index* triglyceride–glucose index; *CI* confidence interval; *NRI* Net reclassification improvement; *IDI* Integrated discrimination improvement, *Ref.* reference

*p* values in bold are < 0.05

## Discussion

With the heavier burdens of metabolic risk factors in Eastern China, this study demonstrated the correlation between the TyG index and incidence of CVD, CHD and stroke in non-diabetic general population for the first time. The risk of CVD and CHD increased significantly with the increase in the TyG index. Furthermore, the addition of the TyG index to the baseline risk model significantly improved its predictive value, thus to better prevent the incidence of CVD and CHD.

IR was believed as an important risk factor for the development of atherosclerosis [14–16] and subsequent adverse cardiovascular events [17, 18]. Given the limitations of traditional assessment methods of IR such as the hyperinsulinemic-euglycemic clamp technique and homeostasis model assessment for IR (HOMA-IR) [19, 20], the TyG index has been evaluated as a simple and reliable surrogate for IR, and has been proven to be associated with CVD events [2, 6, 21, 22].

Previously, results from subgroup analysis of DM on the association between the TyG index and risk of CVD were inconstant. For example, Laura Sánchez-Íñigo et al. found that the TyG index was a risk factor for incident CVD in population without DM, but the TyG index lost its predictive value in population with DM [23]. However, the predictive value of the TyG index was observed only among population with DM in the study conducted by Jiao et al. [24]. In patients with DM, the use of hypoglycemic drugs would affect the actual level of blood glucose, which directly influenced the TyG index. Moreover, in population with DM, traditional cardiovascular risk factors had a greater impact on cardiovascular events than IR [25]. Due to the uncertain predictive value of the TyG index in non-diabetic people, we conducted the study to investigate the association between the TyG index and CVD in population without DM.

Previous studies have found that the TyG index was closely linked to CVD in different countries [26–28]. The studies conducted in North China found that the cumulative TyG index and the change of TyG index were associated with CVD incidence [29, 30]. However, few studies explored the relationship between the TyG index and CVD incidence in eastern China. Eastern China has remarkable differences of living environment, eating habits, social development level, demographic characteristics compared with other regions of the country [31]. In eastern China, with rapid economic development, the metabolic risk factors increased remarkably over the past two decades and the mortality and morbidity of CVD continue to rise at an alarming rate [32]. Due to the heavy burden of metabolic risk factors and the distinct sociodemographic characteristics among eastern Chinese population, we undertook this study.

In the current study, we found that the highest TyG index was associated with the highest incidence of CVD and CHD, which is consistent with the previous reports [2, 6, 23]. However, although the Kailuan study evidenced a positive relationship between the TyG index and stroke [6], we failed to find the significant association in our study. This finding might be due to the facts as follows. Firstly, the population based on the Kailuan community were almost four times as many male as female participants [33] and the stroke incidence in male is higher than that in female in China [34]. Secondly, the Kailuan study also included participants with DM, which may lead to the TyG index an additional effect on stroke. Besides, the information about the intake of fat and carbohydrates, which plays an important role in stroke, was not collected in the Kailuan study.

Subgroup analysis demonstrated that the positive association of the TyG index and CVD/CHD incidence still exited in female, younger than 60 years, without obesity and hypertension participants. Although previous studies showed the predictive values of the TyG index in elderly people for arterial stiffness [35] and CVD [36]. In the current study, we did not find the statistical value in predicting CVD and CHD by the TyG index in subjects aged > 60 years old. Consistent with our finding, a study conducted in Iranians also found that the association between the TyG index and CVD/CHD incidence was more prominent in younger subjects [37]. This may be due to more cardiovascular risk factors in the elderly and thus weaken the predictive value of the TyG index.

There were studies evidenced the improvement for predicting adverse cardiovascular events by adding the TyG index in patients after PCI and elderly ACS patients and patients with PCAD [24, 38, 39]. And we found that the addition of the TyG index to the baseline risk model also significantly improved its predictive value in non-diabetic general population without a history of CVD in Eastern China.

In the analysis of the association between the TyG index and the incidence of CVD, CHD and stroke, we used 6 unadjusted and adjusted models to develop the cox proportional hazard regression. Model 2 only adjusted for demographics (age and sex), which can be ascertained easily, showed great usefulness and generality in clinical practice. Model 3, 5, 6 were partially adjusted. While the fully adjusted model (Model 4) provided the most specific risk prediction model and showed the independent prediction value of the TyG index for the incidence of CVD, CHD and stroke which cannot be explained by other covariates.

Several limitations must also be noted. First, this is an observational study, and we cannot prove causality between the TyG index and incidence of CVD. Second, the TyG index is dynamic and changing, and we do not take into account the changes of TyG index over the follow-up period. Third, our study only investigates the residents in Eastern China, which may not be suitable for the population in other regions. Finally, during the data analysis, we did not divide the secondary endpoint stroke into ischemic stroke and hemorrhagic stroke.

## Conclusion

Our findings suggest that the TyG index is positively associated with the cardiovascular risk factors and can be used to identify individuals at risk of developing CVD in our non-diabetic population cohort. The TyG index may be used as a useful marker for risk stratification in general non-diabetic population.

## Supplementary Information

**Additional file 1: Table S1.** Univariate Cox regression analyses for the incidence of CVD, CHD and stroke.

## Data Availability

The datasets used and/or analyzed during the current study are available from the corresponding author on reasonable request.

## Abbreviations

TyG index: Triglyceride–glucose index
IR: Insulin resistance
CVD: Cardiovascular disease
CHD: Coronary heart disease
CI: Confidence interval
DM: Diabetes mellitus
BMI: Body mass index
WHR: Waist hip ratio
SBP: Systolic blood pressure
DBP: Diastolic blood pressure
EDTA: Ethylene diamine tetraacetic acid
FPG: Fasting plasma glucose
TC: Total cholesterol
TG: Triglyceride
HDL-C: High-density lipoprotein cholesterol
LDL-C: Low-density lipoprotein cholesterol
BP: Blood pressure
SD: Standard deviation
HR: Hazard ratio
NRI: Net reclassification improvement
IDI: Integrated discrimination improvement
HOMA-IR: Homeostasis model assessment for insulin resistance
ACS: Acute coronary syndrome
PCAD: Premature coronary artery disease
