# Supplementary material for: Association of triglyceride–glucose index and traditional risk factors with cardiovascular disease among non-diabetic population: a 10-year prospective cohort study
Source: Cardiovasc Diabetol. 2022 Nov 24;21:256. doi: 10.1186/s12933-022-01694-3 (PMC9700958; doi:10.1186/s12933-022-01694-3)
Supplement: Supplementary file 1 — Additional file 1: Table S1. Univariate Cox regression analyses for the incidence of CVD, CHD and stroke. [file 12933_2022_1694_MOESM1_ESM.docx]

**Table S1 Univariate Cox regression analyses for the incidence of CVD, CHD and stroke**

| Variables | CVD | | CHD | | stroke | |
| --- | --- | --- | --- | --- | --- | --- |
|  | HR(95%CI) | *P*-value | HR(95%CI) | *P*-value | HR(95%CI) | *P*-value |
| Age | 1.066(1.056,1.076) | **<0.001** | 1.062(1.050,1.075) | **<0.001** | 1.071(1.056,1.087) | **<0.001** |
| Gender | 1.122(0.911,1.381) | 0.278 | 0.954(0.734,1.240) | 0.724 | 1.418(1.027,1.958) | **0.034** |
| WHR | 3.030(0.378,24.310) | 0.297 | 4.868(0.375,63.217) | 0.226 | 1.819(0.069,47.833) | 0.720 |
| Tobacco use |  | |  | |  | |
| Never | reference |  | reference |  | reference |  |
| Former | 1.966(1.186,3.259) | **0.009** | 2.457(1.397,4.322) | **0.002** | 1.834(0.805,4.179) | 0.149 |
| Current | 1.297(1.018,1.653) | **0.035** | 1.068(0.773,1.478) | 0.689 | 1.573(1.102,2.247) | **0.013** |
| Alcohol use |  | |  | |  | |
| Never | reference |  | reference |  | reference |  |
| Former | 1.966(1.046,3.698) | **0.036** | 1.811(0.802,4.088) | 0.153 | 3.038(1.333,6.921) | **0.008** |
| Current | 1.264(0.989,1.614) | 0.061 | 1.099(0.798,1.514) | 0.563 | 1.643(1.151,2.347) | **0.006** |
| Education |  | |  | |  | |
| Primary or less | reference |  | reference |  | reference |  |
| Secondary | 0.601(0.485,0.745) | **<0.001** | 0.763(0.579,1.005) | 0.055 | 0.480(0.347,0.664) | **<0.001** |
| Trade, college or university | 0.670(0.426,1.054) | 0.083 | 1.185(0.731,1.922) | 0.491 | 0.130(0.032,0.527) | **0.004** |
| Low physical activity | 1.393(1.013,1.916) | **0.042** | 1.517(1.001,2.299) | **0.049** | 1.327(0.820,2.146) | 0.249 |
| Hypertension | 2.222(1.787,2.762) | **<0.001** | 1.893(1.446,2.478) | **<0.001** | 2.703(1.912,3.823) | **<0.001** |
| BMI | 1.055(1.028,1.082) | **<0.001** | 1.081(1.051,1.112) | **<0.001** | 1.019(0.975,1.064) | 0.411 |
| LDL-C | 1.129(0.983,1.296) | 0.085 | 1.264(1.065,1.501) | **0.007** | 1.043(0.842,1.293) | 0.699 |
| Intake of fat |  | |  | |  | |
| Tertile 1 | reference |  | reference |  | reference |  |
| Tertile 2 | 0.917(0.709,1.186) | 0.509 | 1.029(0.737,1.437) | 0.867 | 0.810(0.551,1.190) | 0.282 |
| Tertile 3 | 1.051(0.819,1.350) | 0.695 | 1.285(0.935,1.767) | 0.122 | 0.807(0.548,1.189) | 0.278 |
| Intake of carbohydrates |  | |  | |  |  |
| Tertile 1 | reference |  | reference |  | reference |  |
| Tertile 2 | 0.747(0.583,0.957) | **0.021** | 0.600(0.441,0.818) | **0.001** | 1.150(0.773,1.711) | 0.490 |
| Tertile 3 | 0.696(0.541,0.897) | **0.005** | 0.488(0.351,0.678) | **<0.001** | 1.146(0.770,1.705) | 0.502 |
| Use of antihypertensive drugs | 2.599(2.020,3.345) | **<0.001** | 2.302(1.655,3.200) | **<0.001** | 2.968(2.041,4.316) | **<0.001** |
| Use of antilipemic drugs | 3.633(2.420,5.456) | **<0.001** | 3.202(1.864,5.501) | **<0.001** | 4.189(2.322,7.555) | **<0.001** |

*p* values in bold are＜0.05

BMI, body mass index; WHR, Waist hip ratio; SBP, Systolic blood pressure; DBP, Diastolic blood pressure; FPG, fasting plasma glucose; TC, total cholesterol; TG, triglyceride; LDL-C, low-density lipoprotein-cholesterol; HDL-C, high-density lipoprotein-cholesterol; CVD, Cardiovascular disease; CHD, Coronary heart disease.
